# Dynamic overrepresentation of accumbal cues in food- and opioid-seeking rats after prenatal THC exposure

**DOI:** 10.1126/sciadv.adq5652

**Published:** 2024-11-08

**Authors:** Miguel Á. Luján, Reana Young-Morrison, Sonia Aroni, István Katona, Miriam Melis, Joseph F. Cheer

**Affiliations:** ^1^ Department of Neurobiology, University of Maryland School of Medicine, Baltimore, MD, USA.; ^2^ Department of Biomedical Sciences, University of Cagliari, Cittadella Universitaria, Monserrato, Italy.; ^3^ Momentum Laboratory of Molecular Neurobiology, Institute of Experimental Medicine, Hungarian Academy of Sciences, Budapest, Hungary.; ^4^ Department of Psychological and Brain Sciences, Indiana University, Bloomington, IN, USA.; ^5^ Department of Psychiatry, University of Maryland School of Medicine, Baltimore, MD, USA.

## Abstract

The increasing prevalence of cannabis use during pregnancy has raised medical concerns, primarily related to Δ9-tetrahydrocannabinol (THC), which readily crosses the placenta and affects fetal brain development. Previous research has identified dopaminergic alterations related to maternal THC consumption. However, the consequences that prenatal cannabis exposure (PCE) has on striatum-based processing during reward pursuit have not been determined. Here, we characterize PCE rats during food or opioid-maintained reward seeking. We find that the supramotivational phenotype of PCE rats is independent of value-based processing and is instead related to augmented reinforcing efficiency of opioid rewards. Our findings reveal that prenatal THC exposure leads to increased cue-evoked dopamine responses and an overrepresentation of effort-driven striatal encoding patterns. Recapitulating clinical findings, drug-related PCE adaptations were more pronounced in males, who showed increased vulnerability for relapse. Collectively, these findings indicate that prenatal THC exposure in male rats engenders a pronounced neurodevelopmental susceptibility to addiction-like disorders.

## Teaser

Prenatal THC exposure heightens addiction vulnerability in male rats, affecting dopamine responses and reward-seeking behavior.

## INTRODUCTION

Use of cannabis during pregnancy is on the rise (*1*, *2*), with an estimated prevalence ranging from 7%, according to self-reports (*3*), to 22.4% when analyzing umbilical cord samples (*4*). Pregnant individuals report using cannabis to relieve stress and anxiety (*5*), a trend worsened by the COVID-19 pandemic (*6*). This alarming trajectory is compounded by misleading marketing strategies by medically licensed dispensaries (*7*) and a growing misconception about the safety of “natural” cannabis plant derivates (*8*). The use of cannabis during pregnancy comes with substantial medical concerns, particularly due to the presence of Δ^9^-tetrahydrocannabinol (THC), the main psychoactive component of cannabis. THC readily crosses the placenta, and a third of plasma content enters the fetus (*9*). Exogenous cannabinoid receptor type 1 (CB1R) agonists, like THC, interfere with endocannabinoid signaling cascades governing progenitor cell proliferation, neuronal differentiation, axon growth, synapse formation and pruning in the developing brain (*10*).

Human longitudinal cohorts have underscored the detrimental effects of prenatal cannabis exposure (PCE), predisposing offspring to a range of neuropsychiatric conditions such as hyperactivity, impulsivity, and increased susceptibility to psychosis (*11*). Rodent studies from our laboratory and others have shed light into the neurobiological disturbances caused by in utero THC exposure, consistently affecting brain dopaminergic pathways along the mesolimbic system (*12*). In the ventral tegmental area (VTA), PCE leads to a maladaptive phenotype characterized by the hyperexcitability of dopamine neurons (*13*–*15*). In the nucleus accumbens (NAc), where VTA dopamine neurons project (*16*), maternal THC exposure induces enduring epigenetic and motivational signatures (*17*). In light of these concerning findings, and human studies disclosing an increased risk of substance use (*18*, *19*), we hypothesize that exposure to THC during pregnancy enhances the reinforcing and dopaminergic effects of drugs of abuse later in life.

To test this possibility, this study examined reward processing and impulsivity. Specifically, we leverage several electrophysiological, optical imaging, and behavioral tools in combination with neuroeconomic and data-driven computational models to parse out aberrant patterns of neuronal encoding and dopamine release in response to natural and opioid reward cues. We demonstrate that distinct endophenotypic motivational traits differentially underlie the enhanced propensity to excessively seek food and drug rewards and confirm that male subjects are at a greater risk of addiction-like vulnerability compared to their female counterparts.

## RESULTS

### Prenatal THC exposure enhances effortful motivation for natural rewards in adult rats

**Fig. 1.**
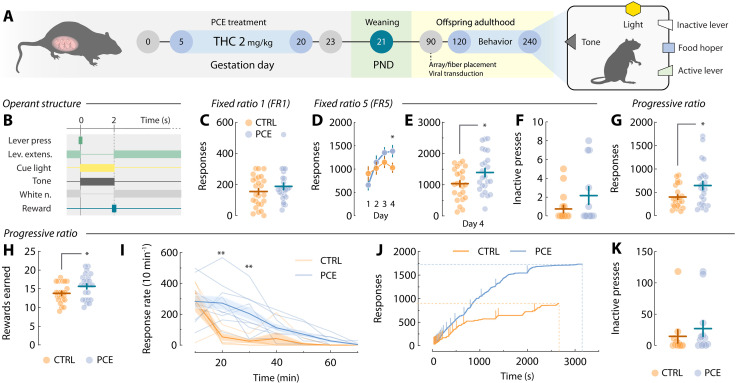
Motivation for food rewards in adult control and PCE progenies. (**A**) Schematic timeline and experimental setup of food self-administration experiments. (**B**) Schematic representation of operant task structure of FR1, FR5, and PR schedules of reinforcement. (**C**) Total active lever presses from the last FR1 training session (*t*_47_ = 1.12 and *P* = 0.26). *n*_CTRL_ = 25 and *n*_PCE_ = 24. (**D**) Average day-by-day active lever presses during FR5 training [two-way repeated-measures (RM) ANOVA; ^treatment^*F*_1,45_ = 1.13 and *P* = 0.29; ^day^*F*_3,120_ = 6.37 and *P* < 0.001; ^interaction^*F*_3,120_ = 3.29 and *P* = 0.023; followed by Bonferroni’s post hoc: day 4, *P = 0.042]. (**E** and **F**) Total active (*t*_45_ = 2.16 and *P = 0.036) and inactive (Welch’s *t*_45_ = 0.95 and *P* = 0.34) lever presses from the last FR5 operant session. *n*_CTRL_ = 23 and *n*_PCE_ = 24. (**G** and **H**) Total active lever presses (Welch’s *t*_35.8_ = 2.36 and *P* = 0.023) and rewards earned (*t*_45_ = 2.1 and *P* = 0.034) under a PR schedule of reinforcement. *n*_CTRL_ = 23 and *n*_PCE_ = 24. (**I**) Average lever pressing frequency during PR responding (two-way RM ANOVA; ^treatment^*F*_1,13_ = 7.48 and *P* = 0.017; ^time^*F*_6,78_ = 22.4 and *P* < 0.001; ^interaction^*F*_6,78_ = 4.24 and *P* < 0.001) (Bonferroni’s post hoc; **P > 0.001). Thick lines and shaded areas represent mean ± SEM values. *n*_CTRL_ = 5 and *n*_PCE_ = 10. (**J**) Representative cumulative responses during PR. Vertical lines denote pellet deliveries. (**K**) Total inactive PR lever presses (*t*_45_ = 0.74 and *P* = 0.46). For all bar and point plots, lines represent mean ± SEM.

To test whether PCE augments motivation for rewards in the adult offspring, we administered the main psychoactive component of the *Cannabis sativa* plant THC (2 mg/kg, s.c., once daily) to rat dams from gestational day (GD) 5 until GD20 (Fig. 1A), a dosing regimen devoid of effects on litter size, maternal care, and offspring body weight (*14*). The vehicle- (CTRL) and THC-exposed (PCE) progeny was left undisturbed until postnatal day (PND) 90, when surgical procedures took place. After surgical recovery, or ~PND120, male and female rats where food restricted and trained to lever press for palatable food rewards under various schedules of reinforcement (Fig. 1B). Results from the fixed ratio 1 (FR1) test indicate no differences due to PCE at low response requirements (Fig. 1C).

Rats then progressed to an FR5 schedule. Under these conditions, PCE offspring showed increased motivation (Fig. 1, D and E), without changes in inactive lever pressing (Fig. 1F). Next, we tested rats on a progressive ratio (PR) schedule, where the response requirement grew exponentially to obtain a single palatable pellet. This measure of effortful motivation was substantially increased in terms of both active lever-pressing (Fig. 1G) and rewards earned (Fig. 1H). Figure 1I depicts average response rates during the PR session, demonstrating higher response vigor in PCE rats compared to control rats. For reference, Fig. 1J displays cumulative response traces from two representative rats during PR. No differences in inactive lever presses were found (Fig. 1K). In addition, no significant effects of sex were found (fig. S1). Together, these results align with clinical findings suggesting a link between PCE and a propensity to manifest enhanced motivation later in life (*11*, *18*). These findings highlight the utility of our rat model to study this clinically relevant phenotype while circumventing residual confounding factors inherent to clinical studies.

### PCE results in a generalized enhancement of cue-evoked dopamine release in the NAc

Vigorous appetitive behaviors triggered by outcome-predictive cues are critically dependent on NAc dopamine release (*20*). Hence, striatal dopamine abnormalities underlie different motivational disorders, such as drug addiction, via dysregulated rewarding behaviors (*21*). We have previously shown that PCE shifts the excitatory-to-inhibitory synaptic input balance onto VTA dopamine neurons (*14*), resulting in increased excitability and firing rates (*13*). However, whether the increase in reward seeking is accompanied by an enhancement of terminal dopamine release in a behaving animal is yet to be determined. Therefore, we used the fluorescent sensor GrabDA_2m_ (*22*) to monitor in vivo NAc dopamine fluctuations during the PR test.

**Fig. 2.**
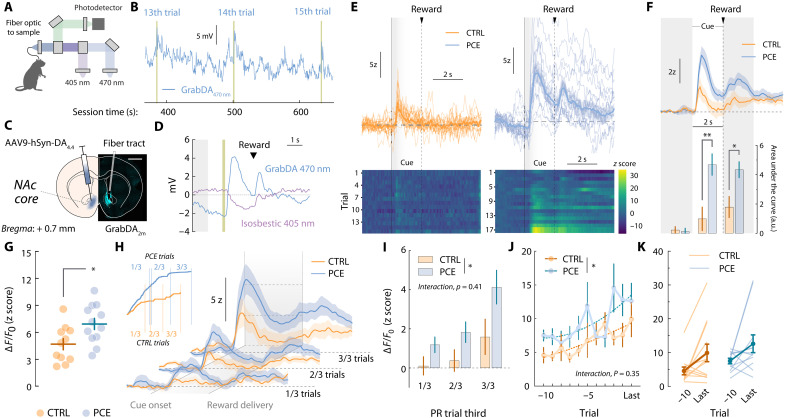
Cue- and reward-evoked NAc dopamine release events during food PR responding. (**A**) Schematic representation of fiber photometry equipment consisting of dopamine-reporting 470-nm-stimulated and artifact-reporting 405-nm-stimulated light signals. (**B**) Raw 470-nm-stimulated stream signal illustrating spontaneous and cue-aligned NAc dopamine transients during PR responding. Yellow bars represent cue presentation. (**C**) Confocal and schematic image showing unilateral injection of the GrabDA_2m_ sensor and fiber tract aimed at the NAc core. Scale bar, 200 μm. (**D**) Representative peri-stimulus time histogram (PSTH) depicting raw GrabDA_2m_ (470 nm) and isosbestic (405 nm) fluctuations time-locked to cue presentation (yellow bar) and reward delivery. Gray shaded area shows baseline period used for *z* scoring. (**E**) Representative heatmap and trial-by-trial dopamine transients during PR responding. Shaded areas (0 to 2 s relative to cue onset) correspond to the time of cue presentation. (**F**) (top) Group-averaged NAc dopamine transients during PR responding. (bottom) Pre-, cue-, and reward-associated dopamine amplitudes (AUC) in control and PCE rats (two-way RM ANOVA; ^treatment^*F*_1,22_ = 5.60 and *P* = 0.027; ^event^*F*_2,44_ = 17.12 and *P* < 0.001; ^interaction^*F*_2,44_ = 5.09 and *P* = 0.010; followed by Bonferroni’s post hoc; **P = 0.009). (**G**) Cue-associated (0 to 2 s relative to cue onset) GrabDA_2m_ peak *z* score values (*t*_22_ = 2.70 and *P = 0.013) during PR. (**H** and **I**) Group-averaged GrabDA_2m_ transients on each PR third. (inset) Representative cumulative responding plot illustrating separation of early (1/3), mid (2/3), and late (3/3) PR sections based on each animal’s total number of completed trials. (**J** and **K**) Cue-associated peak GrabDA_2m_ (*z* scores) values from the last 10 completed PR trials (two-way RM ANOVA; ^treatment^*F*_1,22_ = 4.97 and *P = 0.037; ^trial^*F*_10,220_ = 2.77 and *P* = 0.003; ^interaction^*F*_10,220_ = 1.11 and *P* = 0.35). Vertical lines represent SEM values. For all panels, *n*_CTRL_ = 12 and *n*_PCE_ = 12.

Freely moving rats implanted with optic fibers aimed at the core subfield of the NAc were connected to a fiber photometry system via a patch chord relaying dopamine (470 nm)– and isosbestic (405 nm)–stimulated fluorescence (Fig. 2A). The raw 470-nm-stimulated dopamine signal exhibited fluctuations time-locked to completion of each PR response requirement (Fig. 2B). Postmortem immunohistochemistry confirmed the localized expression of the GrabDA_2m_ probe and the optic fiber placement in the NAc core (Fig. 2C). Potential movement artifacts were subtracted by de-trending isosbestic fluctuations from the 470-nm-stimulated signal (Fig. 2D). Trial-by-trial traces from representative rats depict dopamine encoding of reward-paired cues (Fig. 2E). PCE rats exhibited an increase in the area under the curve (AUC) associated with dopamine fluorescence during the 2-s cue window (Fig. 2F). Figure 2G shows the trial-averaged peak GrabDA_2m_ amplitudes observed during cue presentation, revealing a significant increase of NAc dopamine release triggered by outcome-predictive cues. As shown in fig. S1, no sex-dependent effects of PCE on NAc dopamine release during operant food seeking were observed.

NAc dopamine cue-evoked responses in PCE and control rats increased as a function of response requirement (Fig. 2H) (*23*). Different reasons can account for this behavior, including the signaling of sunk costs (*24*, *25*) and prediction errors (*26*). To explore potential consequences of PCE in these phenomena, we compared cue-evoked GrabDA_2m_ amplitudes obtained during early, middle and late PR trials (Fig. 2, H and I). Although both treatment (^treat^*F*_1,22_ = 6.94 and *P* = 0.015) and trial (^trial^*F*_2,44_ = 10.7 and *P* < 0.001) factors modulated cue-evoked dopamine release, no interaction effects were observed (^interaction^*F*_2,44_ = 0.90 and *P* = 0.41). The lack of interaction between trial (effort level) and THC exposure was additionally confirmed by comparing the last 10 trials ratios completed on the PR session (Fig. 2, J and K). The lack of interaction (^interaction^*F*_10,220_ = 1.11 and *P* = 0.35) between trial (effort level) (^trial^*F*_10,220_ = 2.77 and *P* = 0.003) and treatment (^treat^*F*_1,22_ = 4.97 and *P* > 0.001) supports a baseline propensity for PCE offspring to show greater cue-evoked dopamine responses at all response requirement levels. This vulnerability was independent from potential dopamine fluctuations introduced by sunken cost valuation or error predictions.

### PCE enhances the encoding of cue-responding, effort-related NAc cell assemblies

**Fig. 3.**
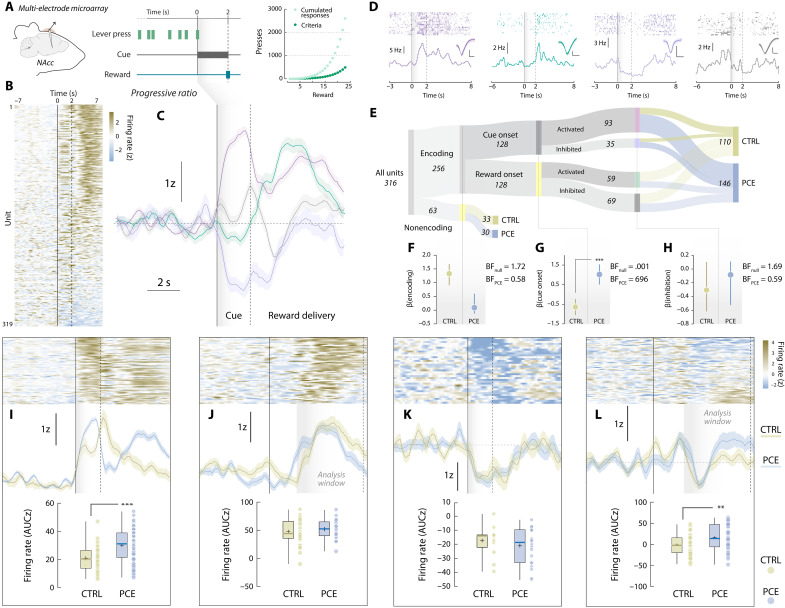
PCE promotes NAc cue-evoked neuronal firing. (**A**) Chronic multi-electrode microarray NAc implantation and PR task structure schematics. (**B**) Trial-averaged, cue-aligned PSTH of all recorded NAc units during PR. (**C**) NAc firing rate patterns uncovered by *k*-means clustering. (**D**) Raster (top) and PSTH (bottom) plots of representative neurons pertaining to each cluster. Insets: average waveforms (scale bars, 50 μV and 20 ms). (**E**) Encoding versus nonencoding, cue onset versus reward onset, and activated versus inhibited units and its treatment assignments. (**F** to **H**) Posterior coefficients (β) with credible intervals (vertical lines) from Bayesian logistic regressions evaluating the influence of PCE on each classification step. Bayes factors (BF) for null and PCE-influenced hypotheses are shown as insets. “***” represents “extreme” evidence in favor of the alternative (PCE-influenced) hypothesis. (**I** to **L**) (Top) Trial-averaged heatmap depicting all neurons’ activity from each cluster. (Middle) Group-averaged NAc firing rate. Vertical solid line: cue onset. Shaded gray areas and vertical dashed lines indicate the time window used for parametric analyses. (Bottom) Trial-averaged firing rates (AUC) during analysis window from the cue-activated (*t*_91_ = 3.64 and ***P > 0.001) (*n*_CTRL_ = 25 and *n*_PCE_ = 68), reward-activated (*t*_57_ = 0.88 and *P* = 0.38) (*n*_CTRL_ = 35 and *n*_PCE_ = 29), cue-inhibited (*t*_33_ = 0.85 and *P* = 0.39) (*n*_CTRL_ = 13 and *n*_PCE_ = 22), and reward-inhibited units (**t_67_ = 2.72 and *P* = 0.008) (*n*_CTRL_ = 37 and *n*_PCE_ = 32). For box plots, the center line represents the median; the cross illustrates the average; the bounds of the box depict the 25th to 75th percentile interval; and the whiskers represent the minima and maxima.

Following these and prior dopaminergic findings (*13*, *14*), we explored potential postsynaptic maladaptations. Using in vivo multiple single-unit electrophysiological recordings in control and PCE rats, we monitored the activity of NAc neurons during PR (Fig. 3A). On the basis of peri-event firing patterns (Fig. 3B), we performed a supervised *k*-means clustering analysis that identified four neuronal responses time-locked to trial completion (Fig. 3C). These clusters were characterized by firing rate increases or decreases at cue (purple and violet) or reward onsets (green and gray) (Fig. 3D) and did not vary as a function of the clustering algorithm applied (fig. S2).

First, we compared the relative abundance of encoding patterns on each treatment condition (Fig. 3E). Bayesian logistic regression models were created at each binary classification step (“encoding versus nonencoding,” “cue onset versus reward onset,” “activated versus inhibited,” and posterior distributions (β) were used to ascertain if the likelihood of finding a biased neuronal classification was higher to that of the null (control) model. Although PCE did not influence the proportion of stimuli-responsive NAc units (Fig. 3F), it biased them to preferentially encode reward-predictive cues instead of rewards (Fig. 3G). PCE, however, did not bias the direction (activation versus inhibition) of the encoding (Fig. 3H). A closer examination revealed that cue-responding neurons from PCE rats fired at higher rates during cue presentation (0 to 2 s) (Fig. 3I). There were no differences in the reward-activated (2 to 7 s) (Fig. 3J) or cue-inhibited (0 to 2 s) (Fig. 3K) clusters; however, the reward-inhibited cluster presented a larger rebound in activity after inhibition (Fig. 3L). These experiments expand NAc dopamine findings by disclosing an accompanying postsynaptic functional adaptation imposed by PCE, causing a disproportional representation of reward-paired cues during operant responding.

**Fig. 4.**
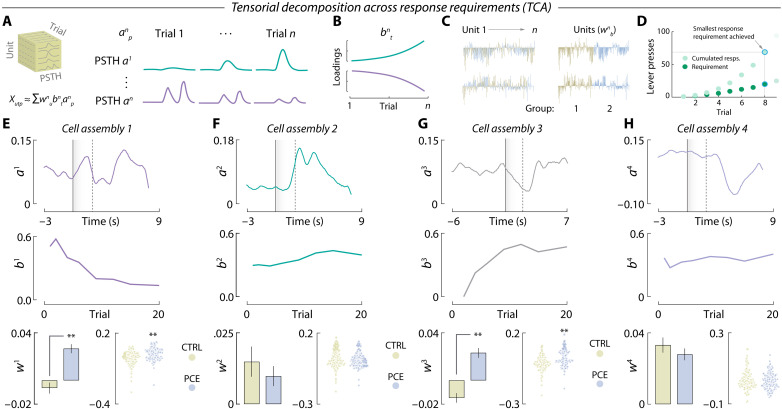
Tensor decomposition reveals overrepresented cue-responding, effort-encoding NAc firing patterns in PCE rats. (**A** and **B**) Tensor representation of trial-structured neural data used to parse out effort-related NAc encoding dynamics during PR responding. (**C**) Example unit factor loadings color-coded by group assignment. In the right, the same units have been ordered by group assignment. (**D**) PR trials, associated lever presses and response requirements selected for TCA analysis. (**E** to **H**) PSTH pattern (top), trial-by-trial temporal evolution (middle), and unit factor loadings (bottom) of the four tensorial cell assemblies identified by TCA. PCE selectively up-regulates effort-related, cue-triggered patterns of neuronal activity in the NAc, as reported by cell assemblies *a*^1^_p_*b*^1^_t_*w*^1^_b_ (**t_317_ = 5.62 and *P* < 0.001) and *a*^3^_p_*b*^3^_t_*w*^3^_b_ (**t_317_ = 6.41 and *P* < 0.001) but not *a*^2^_p_*b*^2^_t_*w*^2^_b_ (Welch’s *t*_254.5_ = 0.79 and *P* = 0.42) and *a*^4^_p_*b*^4^_t_*w*^4^_b_ (*t*_317_ = 0.98 and *P* = 0.32) (*n*_CTRL_ = 143 and *n*_PCE_ = 176).

We next asked whether differential encoding dynamics between treatment groups dynamically diverged as a function of reward cost. To address this question, we used a novel unsupervised algorithm to uncover demixed, low-dimensional neuronal dynamics across multiple timescales (*27*). In brief, we applied tensor component analysis (TCA), which extracts distinct cell assemblies (clusters) from large-scale recordings and groups them based on a three-dimensional low-rank tensor (*28*) reflecting (i) the within-trial peri-event time histogram (PSTH) encoding pattern (*a^n^_p_*, Fig. 4A), (ii) the between-trial temporal evolution of such encoding pattern (*b^n^_t_*, Fig. 4B) and (iii) the contribution of each recorded neuron to a given encoding pattern (*w^n^_b_*, Fig. 4C). Compared to photometry data, multiple single-unit recordings are better suited for TCA but, to normalize the scale of dimension *b^n^_t_* across subjects, we had to restrict our dataset to the first eight trials completed during PR (Fig. 4D), which was the lowest ratio achieved on that session. This allowed us to probe how NAc encoding dynamics evolved across response requirements, encompassing reward costs from 1 to 20 lever presses per pellet. TCA discovered *n* = 4 tensorial cell assemblies, resembling those obtained by *k*-means clustering (Fig. 4, E to H). This approach, however, allowed us to determine that the cue-activated cell assembly (*a*^1^_p_*b*^1^_t_*w*^1^_b_) (Fig. 4E, top) progressively became offline as response requirement increased throughout the PR session (Fig. 4E, middle). Of note, this assembly was more prominent in units recorded from PCE offspring (Fig. 4E, bottom). Figure 4F displays the reward-activated cell assembly *a*^2^_p_*b*^2^_t_*w*^2^_b_ (top), which remained constant across response requirements (middle) and treatment groups (bottom). The cue-inhibited tensor *a*^3^_p_*b*^3^_t_*w*^3^_b_ (Fig. 4G, top), however, progressively became online as effort increased (Fig. 4G, middle) and was again more evident in units recorded from PCE rats (Fig. 4G, bottom). Last, the reward-inhibited cell assembly *a*^4^_p_*b*^4^_t_*w*^4^_b_ (Fig. 4H, top) did not vary throughout the PR session (Fig. 4H, middle) and exhibited similar loadings from control and PCE units (Fig. 4H, bottom). The effects of PCE present in cell assembly loadings were not modulated by sex (fig. S3).

Of note, we asked whether the four tensorial cell assemblies were functionally implicated in the effortful pursuit of food rewards. To do this, we trained a machine learning algorithm (gradient-boosted decision trees) to predict the latency to obtain the eighth PR reward, a behavioral metric not included in the TCA algorithm up until this point. The algorithm was trained on each unit’s loadings to its four tensorial assemblies (*w*^1–4^_b_) and significantly predicted the latency to obtain the eighth reward (ground truth versus predicted value; Pearson’s *r* = 0.44 and *P* = 0.003) (fig. S4).

Together, these results uncover an additional source of alterations in NAc neuronal dynamics after in utero exposure to THC, linking the higher propensity to work harder with an overreactive representation of cues as effort increases. Intriguingly, this dynamic effect contrasts with the monolithic profile of PCE on NAc dopamine release, which did not vary according to reward cost. The combination of these effects points to additional PCE disturbances, beyond or downstream dopamine, in ventral striatal integrative processes regulating effortful motivation across declining rates of reinforcement.

### PCE increases voluntary opioid drug-seeking and relapse in a sex-dependent manner

**Fig. 5.**
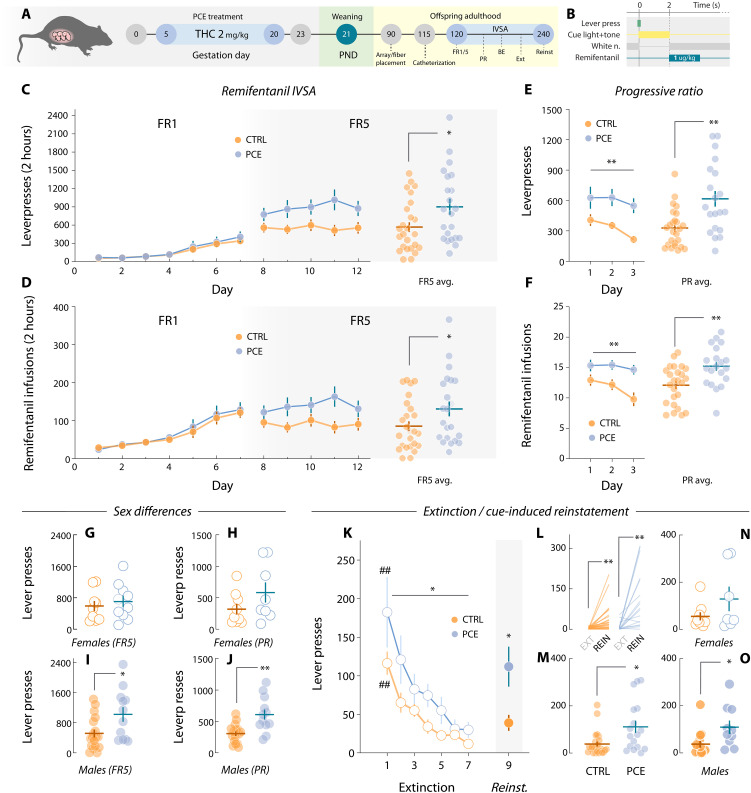
Motivation for drug (remifentanil) rewards in adult PCE rats. (**A**) PCE treatment and remifentanil self-administration schematics. (**B**) Operant task structure. (**C**) Daily active lever presses under FR1 (two-way RM ANOVA; ^treatment^*F*_1,47_ = 0.19 and *P* = 0.66; ^treat·day^*F*_6,265_ = 0.44 and *P* = 0.96) and FR5 schedules of reinforcement (two-way RM ANOVA; ^treatment^*F*_1,47_ = 5.43 and *P* = 0.024; ^treat·day^*F*_4,188_ = 1.98 and *P* = 0.09). The right dot plot shows average FR5 values (*t_47_ = 2.39 and *P* = 0.024). *n*_CTRL_ = 27 and *n*_PCE_ = 22. (**D**) Daily remifentanil infusions obtained under FR1 (two-way RM ANOVA; ^treatment^*F*_1,47_ = 0.07 and *P* = 0.77; ^treat·day^*F*_6,262_ = 0.53 and *P* = 0.77) and FR5 (two-way RM ANOVA; ^treatment^*F*_1,47_ = 4.11 and *P* = 0.048; ^treat·day^*F*_4,188_ = 2.14 and *P* = 0.07). The right dot plot shows average FR5 infusions (*t_47_ = 2.06 and *P* = 0.048). *n*_CTRL_ = 27 and *n*_PCE_ = 22. (**E**) Daily (two-way RM ANOVA; ^treatment^*F*_1,43_ = 12.5 and **P = 0.001; ^treat·day^*F*_2,86_ = 0.54 and *P* = 0.58) and average (Welch’s **t_27.9_ = 3.35 and *P* = 0.002) lever presses during PR testing. (**F**) Daily (two-way RM ANOVA; ^treatment^*F*_1,39_ = 8.11 and **P = 0.007; ^treat·day^*F*_2,78_ = 3.83 and *P* = 0.025) and average (**t_39_ = 2.85 and *P* = 0.007) PR remifentanil rewards. *n*_CTRL_ = 25 and *n*_PCE_ = 20. (**G** and **H**) Female rat lever pressing levels during FR5 (*t*_17_ = 0.58 and Bonferroni-Dunn-corrected *P* = 0.99) (females, *n*_CTRL_ = 9 and *n*_PCE_ = 10) and PR sessions (*t*_15_ = 1.50 and Bonferroni-Dunn-corrected *P* = 0.30) (females, *n*_CTRL_ = 9 and *n*_PCE_ = 8). (**I** and **J**) Average male lever pressing levels during FR5 (**t_26_ = 3.11 and Bonferroni-Dunn-corrected *P* = 0.008) (males, *n*_CTRL_ = 18 and *n*_PCE_ = 12) and PR (**t_24_ = 3.87 and Bonferroni-Dunn-corrected *P* = 0.001) (males, *n*_CTRL_ = 16 and *n*_PCE_ = 12). (**K**) Average lever presses during extinction (two-way RM ANOVA; ^treatment^*F*_1,39_ = 5.27 and *P = 0.027; ^treat·day^*F*_6,232_ = 1.18 and *P* = 0.31; followed by Bonferroni’s post hoc; CTRL day 1 versus day 7, ^##^*P* < 0.001; PCE day 1 versus day 7, ^##^*P* < 0.001) and reinstatement (Welch’s *t_23_ = 2.60 and *P* = 0.016). (**L**) Within-subject lever pressing change from last extinction day (EXT) to reinstatement test (REIN) in control (paired *t*_23_ = 3.33 and Bonferroni-Dunn-corrected **P = 0.005) and PCE rats (paired *t*_16_ = 3.84 and Bonferroni-Dunn-corrected **P = 0.002). *n*_CTRL_ = 24 and *n*_PCE_ = 17. (**M**) Lever presses during the cue-induced reinstatement test (Welch’s *t_21.3_ = 2.60 and *P* = 0.016). (**N**) Female remifentanyl-seeking lever presses during reinstatement (*t*_14_ = 1.48 and Bonferroni-Dunn-corrected *P* = 0.31). Females, *n*_CTRL_ = 9 and *n*_PCE_ = 7. (**O**) Male reinstatement lever presses (*t*_23_ = 2.57 and Bonferroni-Dunn-corrected *P = 0.034). Males, *n*_CTRL_ = 15 and *n*_PCE_ = 10.

Clinical cohorts and longitudinal studies point to an increased risk of substance use in adolescent and young adults prenatally exposed to cannabis (*18*, *19*, *29*). This phenomenon has been replicated in PCE rodents, which exhibit marked behavioral adaptations when exposed to drugs of abuse later in life (*30*–*33*). Notably, the risk of subsequent drug use (in this case, cannabis) was higher for male individuals relative to their female counterparts (*18*). This agrees with the hyperdopaminergic findings described in male PCE rats (*14*). With this evidence at hand, as well as the in vivo dopaminergic and food reward processing disturbances described here, we determined whether effortful motivation for a drug (opioid) reward was also enhanced, and whether potential sex differences could arise when pursuing drug reinforcers. Control (*n* = 27) and PCE (*n* = 22) male and female adult rats were subjected to jugular vein catheterization surgery to allow for voluntary intravenous infusions of the fast-acting fentanyl analog remifentanil (1 μg/kg per infusion) (Fig. 5A). We chose remifentanil for its short half-life, which clears from blood in ~0.3 and ~10 min from the NAc (*34*). Its rapid clearance reduces the risk of overdose, rendering it ideal for rodent intravenous self-administration (IVSA) studies (*35*) and within-session behavioral economics tests (see below) (*36*). This, in combination with its abuse potential in humans (*37*), makes remifentanil a safe, convenient, and relevant tool for rodent studies on opioid use.

As with food rewards, remifentanil infusions (2 s) were preceded by a 2-s multisensorial compound cue (light + tone) (Fig. 5B). Both control and PCE rats learned to lever press at similar rates (Fig. 5C). On FR5, PCE rats started to exhibit increased levels of lever pressing (Fig. 5C). Figure 5D shows drug infusions obtained throughout FR1 and FR5, also significantly different. Next, rats underwent three IVSA PR sessions, in which PCE rats displayed higher levels of effortful motivation to work for remifentanil in terms of total lever presses (Fig. 5E) and drug infusions (breakpoints) (Fig. 5F). Potential sex differences in these metrics were explored by comparing sex-matched subjects across treatments. No significant differences in FR5 (Fig. 5G) and PR lever pressing (Fig. 5H) were found among females. However, in accordance with clinical evidence (*18*), male PCE offspring displayed higher levels of remifentanil seeking during FR5 (Fig. 5I) and PR sessions (Fig. 5J). Overall, results confirm that the hypermotivational phenotype associated with PCE extends to drug rewards, in particular the synthetic opioid remifentanil. Unaffected FR1 performance suggests, once again, that no gross learning effects could explain the observed changes in motivation. Unlike with natural rewards, the consequences of PCE in remifentanil-seeking rats were influenced by sex, conferring a higher vulnerability to the male offspring, and recapitulating the clinical phenotype.

Persisting risk of relapse remains a defining feature of substance use disorders and the primary end point for treatment considerations (*38*, *39*). Owing to the increase in opioid intake and seeking uncovered here, we next aimed to parse out PCE effects on remifentanil seeking in drug-free conditions (extinction) and upon re-exposure to drug-paired cues (reinstatement), a rodent model of relapse (*40*). PCE rats showed increased overall drug seeking levels during extinction, but the rate at which they ceased seeking the drug-paired lever was not different (Fig. 5K). Upon reintroduction of drug cues, both control and PCE rats increased their respective levels of opioid-seeking compared to the last extinction session (Fig. 5L). Notably, PCE rats exhibited greater levels of cue-induced reinstatement (Fig. 5, L and M). We did not detect this increase when comparing females across treatments (Fig. 5N). However, male PCE offspring showed a marked increase in cue-induced reinstatement when compared to male control rats (Fig. 5O).

### PCE exacerbates opioid-induced dopamine release in the NAc of PCE animals

**Fig. 6.**
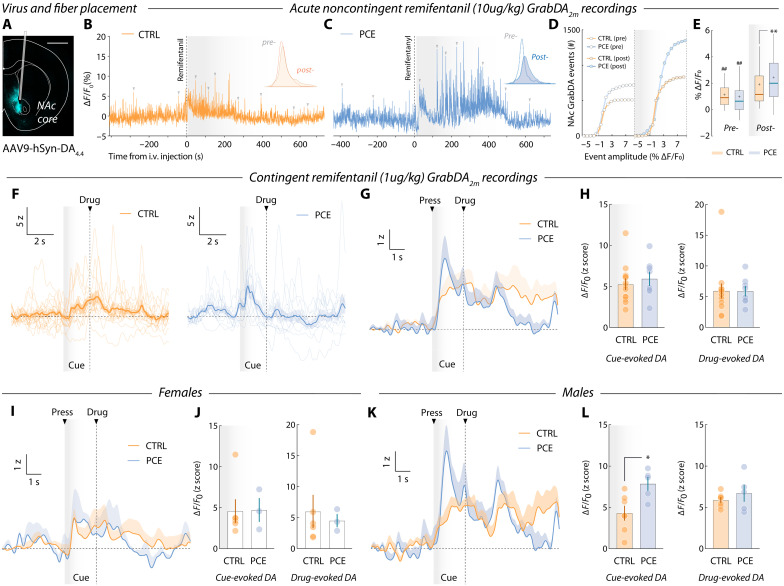
NAc dopamine release dynamics during remifentanil injection and voluntary intake. (**A**) Representative image showing viral expression localization and optic fiber placement in the NAc core. (**B** and **C**) Spontaneous GrabDA_2m_ transients (indicated with gray triangles) before and after remifentanil intravenous injection by experimenter. Insets illustrate distributions of spontaneous event amplitudes (% Δ*F*/*F*_0_) pre-(white) and postinjection (colored). (**D**) Cumulative amplitude distribution of NAc dopamine release events before and after remifentanil administration. (**E**) Pre-post injection comparison of NAc dopamine events from control and PCE rats (two-way RM ANOVA; ^treatment^*F*_1,3462_ = 4. 37 and *P* = 0.030; ^remifentanil^*F*_1,3462_ = 176.8 and *P* < 0.001; ^interaction^*F*_1,3462_ = 85.5 and *P* < 0.001; followed by Bonferroni’s post-hoc: pre-post CTRL, ^##^*P* < 0.001; pre-post PCE, ^##^*P* < 0.001; post-, CTRL versus PCE, ^**^P < 0.001). *n*_CTRL_ = 5 and *n*_PCE_ = 4. Center line represents the median; the cross illustrates the average; the bounds of the box depict the 25th to 75th percentile interval; and the whiskers represent minima and maxima. (**F**) Representative trial-by-trial and average NAc dopamine transients in response to drug-paired cue presentation during PR. (**G**) Group-averaged NAc dopamine transients. (**H**) Cue- (0 to 2 s, relative to cue onset) (*t*_19_ = 0.63 and *P* = 0.53) and drug-evoked (2 to 8 s, relative to cue onset) (*t*_19_ = 0.02 and *P* = 0.97) dopamine amplitudes (AUC) in control and PCE rats (*n*_CTRL_ = 13 and *n*_PCE_ = 8). (**I** and **J**) Group-averaged NAc dopamine PSTHs from control and PCE female animals (cue-evoked amplitudes: *t*_7_ = 0.05 and Bonferroni-Dunn-corrected *P* > 0.99) (drug-evoked amplitudes: *t*_7_ = 0.37 and Bonferroni-Dunn-corrected *P* > 0.99). Females, *n*_CTRL_ = 6 and *n*_PCE_ = 3. (**K** and **L**) Group-averaged NAc dopamine PSTHs from control and PCE male rats (cue-evoked amplitudes: *t*_10_ = 2.89 and Bonferroni-Dunn-corrected *P* = 0.031) (drug-evoked amplitudes: *t*_10_ = 0.91 and Bonferroni-Dunn-corrected *P* = 0.76). Males, *n*_CTRL_ = 7 and *n*_PCE_ = 5. For all bar and point plots, bars denote mean ± SEM.

The reinforcing potential of abuse drugs depends on its ability to increase the concentration of dopamine in certain brain structures (*41*, *42*), such as the ventral striatum (*43*). To probe drug-evoked dopaminergic alterations imposed by PCE, we implanted optic fibers and the GrabDA_2m_ sensor in the NAc of remifentanil-seeking rats (Fig. 6A).

First, drug-naïve, catheterized rats were left undisturbed in an operant chamber (no manipulanda available) for 10 min before receiving a noncontingent high-dose remifentanil infusion (10 μg/kg). Spontaneous NAc GrabDA_2m_ fluctuations were recorded for 15 min and dopamine release events were identified based on their prominence (Materials and Methods) (Fig. 6, B and C). Pre- and postdrug release events were distributed on the basis of their amplitudes (Fig. 6, B and C, insets). Results indicate that, while both groups exhibited larger-amplitude NAc dopamine release events postdrug infusion, PCE amplified this response (Fig. 6, D and E), revealing a neuropharmacological propensity to experience larger dopaminergic responses to remifentanil.

We then assessed whether PCE enhances the encoding of drug-paired cues during voluntary pursuit of remifentanil. Control and PCE animals were recorded during PR responding. Trial-by-trial representative recordings reveal phasic GrabDA_2m_ transients time-locked to the onset of the drug-paired cues (Fig. 6F). Group-averaged measurements confirm this pattern of encoding (Fig. 6G), but do not disclose differences in the detected peak amplitudes during the cue (0 to 2 s) and drug infusion (2 to 8 s) time windows (Fig. 6H). Despite this, further analysis revealed sex-specific differences. Accordingly, no differences in cue- or drug-evoked dopamine encoding were found among female control and PCE rats (Fig. 6, I and J). Notably, a specific increase in dopaminergic encoding of opioid-paired cues was found among PCE males (Fig. 6, K and L). Of note, the male-specific enhancement in opioid cue encoding agrees with the previously disclosed PCE male willingness to work harder for remifentanil.

### Male PCE offspring NAc neurons disproportionately signal drug-paired cues

**Fig. 7.**
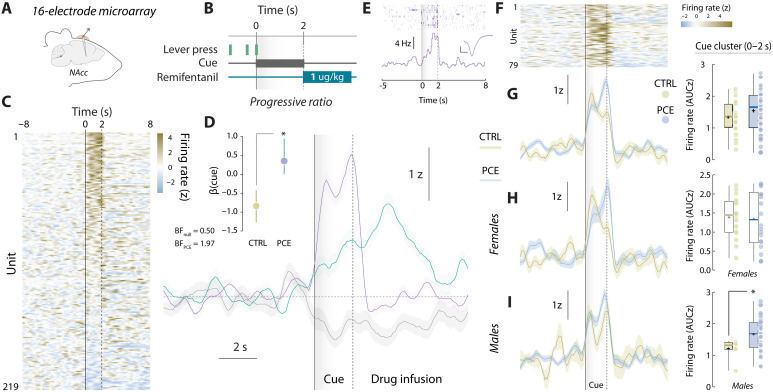
NAc encoding of drug-paired cues during voluntary remifentanil pursuit. (**A** and **B**) Schematic representation of chronic multi-electrode microarray NAc implantation and PR operant task structure. (**C**) Trial-averaged, cue-aligned PSTHs of all NAc units recorded during PR responding. (**D**) Trial-averaged firing rates from the cue-responding (*n*_CTRL_ = 23 and *n*_PCE_ = 56), reward-responding (*n*_CTRL_ = 23 and *n*_PCE_ = 39), and lever press-inhibited clusters units (*n*_CTRL_ = 31 and *n*_PCE_ = 23). (Inset) Posterior coefficients (β) with credible intervals (vertical lines) of Bayesian logistic regression models evaluating the influence of PCE on the likelihood to find a unit on the cue-responding cluster. Bayes factors (BF) for null and PCE-influenced hypotheses are shown as insets. “*” represents “anecdotic” evidence in favor of the alternative (PCE-influenced) hypothesis. (**E**) Raster (top) and PSTH (bottom) plot of a representative neuron pertaining to the cue-responding cluster. Inset shows the corresponding waveform (scale bars, 50 μV and 20 ms). (**F**) Trial-averaged heatmap depicting all neurons’ activity from the cue-responding cluster. (**G**) (Left) Trial-averaged PSTH of the cue-responding cluster in control and PCE rats. (Right) Average firing rates (AUC) during cue presentation (gray shaded area, 0 to 2 s) from cue-responding units (*t*_77_ = 1.36 and *P* = 0.17). (**H** and **I**) (Left) Cue-responding cluster neurons average firing rates from female and male animals. (Right) Average firing rates (AUC) during cue presentation from cue-responding female (*t*_36_ = 0.21 and Bonferroni-Dunn-corrected *P* > 0.99) and male neurons (*t_39_ = 3.01 and Bonferroni-Dunn-corrected *P* = 0.018). *n*_CTRL_ = 23 (16 F and 7 M) and *n*_PCE_ = 56 (22 F and 34 M). Center line represents the median; the cross illustrates the average; the bounds of the box depict the 25th to 75th percentile interval; and the whiskers represent minima and maxima.

We next evaluated whether ventral striatal neuronal firing dynamics were similarly disturbed. In this case, a subset of control and PCE rats underwent chronic multi-electrode implantation surgery (Fig. 7, A and B) and their NAc neuronal responses were monitored during voluntary pursuit of remifentanil (PR) (Fig. 7C). The same *k*-means clustering algorithm used on the food PR dataset was applied, this time identifying three distinct modes of firing in response to trial completion (Fig. 7D). We found that NAc units either preferentially increased their firing rates during cue presentation (0 to 2 s relative to cue onset) or remifentanil infusion (2 to 7 s relative to cue onset). A third, more modest subpopulation started inhibiting its firing rates before the preceding lever press. To detect potential encoding biases introduced by PCE, we performed a Bayesian logistic regression model comparing the relative proportion of NAc units pertaining to the cue-responding cluster. The likelihood of finding a PCE unit in the cue-responding cluster was higher than that observed for control units (Fig. 7D, inset), confirming that drug-paired cues become overrepresented in functionally defined subpopulations of the NAc.

We next focused our attention on the cue-responding subpopulation (Fig. 7, E and F). To compare the relative strength of encoding within this cell assembly, we obtained normalized firing rates (AUC) during the cue presentation time window (0 to 2 s relative to cue onset) and found no gross differences between control and PCE rats (Fig. 7G). However, when we segregated firing rate data by sex to look for a potential male vulnerability, we found that female PCE offspring neurons fire at similar rates than their sex-matched control counterparts (Fig. 7H). Cue-responding NAc neurons of male PCE rats fired at considerably higher rates (Fig. 7I), further supporting the idea that adult male PCE rats are especially vulnerable to the reinforcing, dopaminergic, and striatal actions of remifentanil. No differences were found on the other two clusters (fig. S5).

### Distinct reward processing endophenotypes underlie PCE-induced excessive motivation

Increased reward-directed behaviors for food and remifentanil (1), augmented cue-evoked dopamine release (2), and subsequent effort-related NAc neuronal dynamics (3) all accompany a motivational endophenotype likely predisposing PCE individuals to drug addiction. Nonetheless, these three outcomes can also derive from a more parsimonious, nonpathological neuropsychological adaptation. That is, should PCE animals experience greater hedonic responses to appetitive rewards, adaptations 1 to 3 would be interpreted beneficial, as they would ensure the consecution of higher-valued goods. Resolving this conundrum is crucial to avoid mistaking a vulnerability to drug seeking for a more innocuous hedonic value-seeking phenotype.

**Fig. 8.**
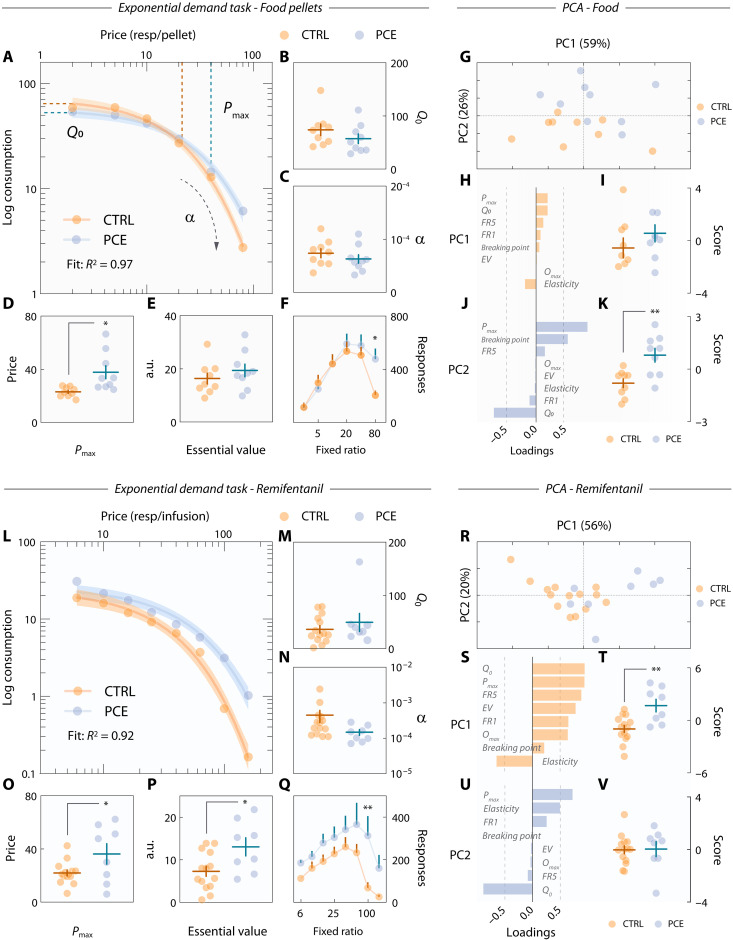
Behavioral economics modeling of food and remifentanil demand. (**A**) Exponential demand curve for food rewards. Shaded areas represent 95% CI. (**B**) *Q*_0_ in control and PCE rats (*t*_16_ = 1.21 and *P* = 0.24). (**C**) PCE does not influence price sensitivity (α) (*t*_16_ = 0.94 and *P* = 0.36). (**D**) *P*_max_ in control and PCE rats (Welch’s *t_9.0_ = 2.92 and *P* = 0.017). (**E**) Unchanged food essential value in control and PCE rats (*t*_16_ = 0.97 and *P* = 0.34). (**F**) Session-averaged lever press responses at each price point (two-way RM ANOVA; ^treatment^*F*_1,16_ = 0. 82 and *P* = 0.37; ^price^*F*_5,80_ = 28.5 and *P* < 0.001; ^interaction^*F*_5,80_ = 3.13 and *P* = 0.012; Bonferroni’s post hoc: CTRL versus PCE, *P = 0.013). (**G**) PC1 (“inelastic lever pressing”) and PC2 (“anhedonic motivational drive”) scores. Percentages indicate the proportion of total behavioral variance explained by each factor. (**H**) Variable loadings onto PC1. (**I**) Inelastic lever pressing (PC1) scores (*t*_16_ = 0.97 and *P* = 0.34). (**J**) Variable loadings onto PC2. (**K**) Anhedonic motivational drive (PC2) scores (**t_16_ = 3.46 and *P* = 0.003). For all food pellet panels, *n*_CTRL_ = 9 and *n*_PCE_ = 9. (**L**) Exponential demand curve for remifentanil. Shaded areas represent 95% confidence interval. (**M** to **P**) *Q*_0_ (*t*_20_ = 0.85 and *P* = 0.40), α (Welch’s *t*_13.6_ = 1.78 and *P* = 0.097), *P*_max_ (*t_20_ = 2.23 and *P* = 0.037), and essential value (*t_20_ = 2.57 and *P* = 0.018) derived from remifentanil demand curves. (**Q**) Session-averaged lever presses for remifentanil at each price point (two-way RM ANOVA; ^treatment^*F*_1,20_ = 4.64 and *P* = 0.043; ^price^*F*_7,140_ = 13.7 and *P* < 0.001; ^interaction^*F*_7,140_ = 2.37 and *P* = 0.025; Bonferroni’s post hoc: CTRL versus PCE, **P = 0.001). (**R**) Individual scores on the two principal components identified by PCA in the remifentanil dataset. (**S**) Variable loadings onto PC1. (**T**) Inelastic lever pressing for remifentanil (**t_20_ = 3.53 and *P* = 0.002). (**U**) Variable loadings onto PC2. (**V**) Anhedonic motivational drive of remifentanil demand (*t*_20_ = 0.09 and *P* = 0.92). For all drug-related panels, *n*_CTRL_ = 14 and *n*_PCE_ = 8. For all bar and point plots, lines denote mean ± SEM.

We sought to resolve this ambiguity by exploiting neuroeconomic modeling tools on an exponential demand task (*44*). After PR testing, food-seeking control and PCE rats progressed to the demand task. During five 50-min-long sessions, response requirements (or “price”) progressively increased every 10-min and averaged demand levels (pellets obtained) per price point were fitted using a neuroeconomic exponential demand equation (*45*) (Fig. 8A). The parameters *Q*_0_, α, *P*_max_, and EV were then used to map distinct reward valuation processes as a function of PCE. *Q*_0_ is denoted as the reward quantity consumed at zero price (Fig. 8A, *y* intercept) and corresponds to the reward’s hedonic set point (*46*, *47*). Control and PCE animals shared *Q*_0_ levels (Fig. 8B). Unaffected *Q*_0_ values indicate that the palatability of food pellets was the same for PCE rats and, therefore, the hypermotivational and hyperdopaminergic phenotypes described must derive from other sources of reward valuation. One alternative is price sensitivity (*48*), which is denoted by α and is orthogonal to *Q*_0_. In this case, increased effortful motivation would be explained by decreased sensitivity (α) to price increments (i.e., response requirements), making rats insensitive to increasing difficulty and displaying greater breakpoints. However, α values of PCE rats were equivalent to those of control subjects (Fig. 8C). A third possibility is an increase in the inflection point between inelastic and elastic demand, represented by *P*_max_. *P*_max_ is related to α and indicates the threshold value (in response requirement) at which price sensitivity sets in. In other words, *P*_max_ coincides with the peak of responding rates and is interpreted as motivational drive; that is, the maximum amount of behavioral resources a subject is willing to allocate to obtain a standard-valued reward (*47*). We found that PCE animals exhibited much larger *P*_max_ values (Fig. 8D), thereby supporting an excessive motivational drive that is independent of hedonic set points (*Q*_0_) and sensitivity to declining opportunity costs (α). Figure 8E shows that the essential value of food pellets, its reinforcing efficiency at all price points, was unaffected. Last, Fig. 8F contains the raw demand task lever pressing data from which all parameters were derived.

Next, we performed a dimensionality reduction in all behavioral measurements obtained (all neuroeconomic parameters, FR1–5, and PR) to gain access to a more generic manifestation of the underlying motivational endophenotype (*49*). Principal components analysis (PCA) identified two low-dimensional factors explaining 84.7% of all behavioral variance (Fig. 8G). The first principal component (PC1) reflected inelastic lever pressing, as all variables related to lever pressing (*P*_max_, *Q*_0_, FR5 responses, and PR rewards earned) loaded positively onto this dimension, while elasticity (α) loaded negatively (Fig. 8H). Both control and PCE rats scored similarly in this dimension (Fig. 8I), confirming that this food-seeking behavioral signature was not affected. A second dimension, PC2, positively related *P*_max_ and PR rewards earned with small *Q*_0_ (Fig. 8J), indicative of “anhedonic” motivational drive. It is this endophenotype that clearly characterized the motivational disturbances regarding natural rewards, as THC-exposed rats scored much higher in this dimension (Fig. 8K). Together, these results confirm that adult PCE offspring suffer from excessive motivation for natural rewards, even when these are less valued and in absence of benefit-cost calculations.

Because of their pharmacological effects (*20*) and the lack of homeostatic compensatory mechanisms (*50*), drugs of abuse function differently compared to natural rewards (i.e., food pellets). Therefore, to investigate further signs of maladaptive behavior related to addiction-like behaviors, we conducted the same endophenotypic analysis on remifentanil self-administering rats. Following PR testing, and before extinction sessions, control and PCE rats conducted five 80-min-long remifentanil exponential demand tasks (Fig. 8L). As with natural rewards, PCE augmented maximum motivational drive (*P*_max_) for remifentanil but did not influence its related hedonic set point (*Q*_0_) or price sensitivity (α) (Fig. 8, M to O). In addition, remifentanil had a higher essential value (Fig. 8P). The essential value of a reward reflects its reinforcing efficiency at all price points and can be clearly visualized in the generalized increase in lever pressing observed during the demand task (Fig. 8Q). Principal component analysis revealed two low-dimensional signatures (Fig. 8, R to V), resembling those of food seeking behavior. Intriguingly, when working for a drug reward, PCE induced a specific increase in inelastic lever pressing (PC1) (Fig. 8T) but not “anhedonic” motivational drive (PC2) (Fig. 8V). Together, these results demonstrate that distinct endophenotypic alterations underlying seemingly equivalent increases in natural and drug reward-seeking behaviors are a hallmark of PCE.

### PCE rats exhibit higher impulsivity

**Fig. 9.**
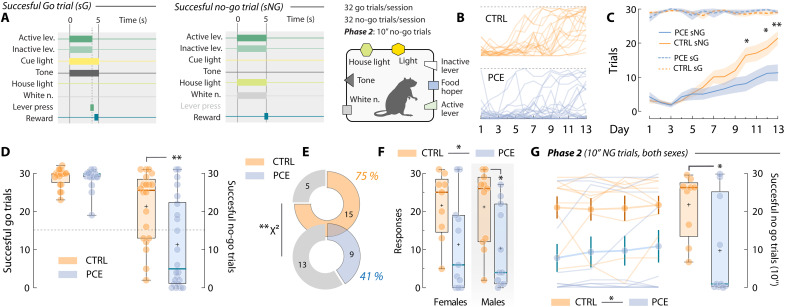
PCE increases impulsive behavior in a go/no-go task. (**A**) Schematic illustrating the compound cues and levers available in the operant chamber during intertrial intervals, as well as the consequences (pellet delivery) of successfully executed go and no-go trials. (**B**) Individual daily progression of successful no-go responding in control and PCE rats. (**C**) Group-averaged number of successfully executed go (sG, dashed lines) and no-go (sNG, solid lines) trials (two-way RM ANOVA; ^treatment^*F*_1,40_ = 3.36 and *P* = 0.07; ^day^*F*_12,480_ = 29.6 and *P* < 0.001; ^interaction^*F*_12,480_ = 4.65 and *P* < 0.001; followed by Bonferroni’s post hoc: CTRL versus PCE; day 10 *P = 0.016, day 12 *P = 0.016, and day 13 **P = 0.001). (**D**) Successfully executed go (*t*_40_ = 0.17 and *P* = 0.86) and no-go trials (**t_40_ = 3.07 and *P* = 0.003) after training (day 13). Dashed line marks 50% performance. (**E**) Percentage of control and PCE rats obtaining more than 50% of no-go trial rewards (χ^2^ = 61.6 and **P < 0.001). (**F**) Successfully executed no-go trials by sex (females; *t*_18_ = 2.11 and Bonferroni-Dunn-corrected *P* = 0.097) (males; *t*_20_ = 2.47 and Bonferroni-Dunn-corrected *P = 0.045). *n*_CTRL_ = 20 (9 F and 11 M) and *n*_PCE_ = 22 (11 F and 11 M). (**G**) Daily (left) and average (right) successfully executed 10” no-go trials in phase 2 (*t_17_ = 2.38 and *P* = 0.029) (*n*_CTRL_ = 9 and *n*_PCE_ = 10) (both sexes). For all box plots, center line represents the median; the cross illustrates the average; the bounds of the box depict the 25th to 75th percentile interval; and the whiskers represent minima and maxima.

Impulsivity deficits are one of the outcomes most consistently associated with PCE (*51*–*55*) and are hypothesized to underlie increased risk of substance use observed after PCE (*11*). Thus, a failure in inhibitory control required to interrupt or suppress highly automatized reward-seeking behaviors could contribute to the PCE hypermotivational phenotype (*56*). Hence, to examine whether PCE rats also presented heightened impulsive actions, we trained rats on a go/no-go task (*57*). Go trials were signaled by a 5-s compound cue (light + tone) and a lever press within cue presentation earned a palatable food pellet reward (Fig. 9A). No-go trials were signaled by a different 5-s compound cue (house light + white noise) and were rewarded only if no lever press was detected during cue presentation (Fig. 9B). Control and PCE rats were trained on this task during 13 sessions (Fig. 9C). As all rats flawlessly executed nearly all go trials (Fig. 9D), the number of successfully executed no-go trials per session was considered a measure of each animal’s inhibitory self-control. Data from the final session, when impulsivity levels were overall lower, revealed that PCE rats displayed higher levels of impulsivity (Fig. 9, D and E). In line with the male-specific PCE impairments examined so far, no significant differences in no-go performance were present among control or PCE female subjects (Fig. 9F). However, impulsivity differences reached statistical significance when control males were compared against PCE male subjects (Fig. 9F). Last, a subset of rats progressed to a second phase of higher difficulty, in which lever pressing had to be withheld for 10 s (Fig. 9G). A significant difference between control and PCE rats (both sexes included) was also found at this level of inhibitory control (Fig. 9G). Therefore, we reveal another maladaptive trait associated to PCE and related to the ability to control highly automatized reward-oriented motor actions (*58*).

## DISCUSSION

In the present study, we provide evidence that prenatal exposure to THC leads to modifications in striatum-based processing consistent with an increased risk for opioid reinforcement and, ultimately, relapse. These alterations encompass an increased willingness to work for natural and drug rewards, up-regulated dopamine release responses to outcome-predictive cues, overrepresentation of effort-encoding NAc neural dynamics, and elevated impulsivity. Collectively, these consequences of PCE correspond to well-established neurodevelopmental risk factors for drug use disorders (*59*, *60*).

Individual differences contribute to the development of long-term maladaptations driving compulsive drug use and relapse propensity (*61*). Maternal exposure to THC is such a predictive risk factor (*13*). Nonetheless, little is known about the neuropsychological constructs leading to this propensity. In our study, we present neuroeconomical evidence of a detrimental trait influencing how PCE individuals work for natural and drug reinforcers. On one hand, the increased willingness of PCE offspring to work impulsively for natural (food) rewards is associated with their intrinsically higher motivational drive, even when accounting for individual differences in reward valuation. On the other hand, increased remifentanil-seeking is a manifestation of a greater susceptibility to the reinforcing actions of opioids, independent of their hedonic effects. In terms of Berridge and Robinson’s (*62*) framework of addiction (*63*), these observations support the hypothesis of a hypersensitized “wanting” system, in contrast to reward valuation (“liking”) or learning alterations, which we [and others (*17*)] demonstrate are not implicated in the motivational phenotype of PCE. A specific increase in incentive salience provides a satisfactory explanation for this and prior rodent studies reporting enhanced effortful motivation and psychomotor activation (*64*) in response to heroin (*30*, *65*), food (*17*), THC (*14*), and alcohol (*66*) [reviewed in (*11*)].

Preclinical and clinical evidence have established a causative role for enhanced mesostriatal dopamine function in the attribution of incentive salience and, ultimately, the pathogenesis of addiction (*67*, *68*). Accordingly, we report that augmented NAc dopamine release is a hallmark of PCE. This finding expands previous evidence indicating that VTA dopamine cells become hyperexcitable following maternal THC exposure (*14*) and other reports showing increased drug-evoked dopamine synthesis (*15*). In addition, our photometry readouts reveal a specific up-regulation of dopamine release in response to reward-paired cues, a dysregulation known to predispose subjects to compulsively seek drugs of abuse (*69*, *70*) and a higher risk of relapse (*20*). Specifically, reintroduction of opioid-paired cues triggered greater remifentanil-seeking. Related to this, PCE rats exhibited signatures of hyper-responsive NAc dopamine activity, even in drug-naïve conditions. It has been proposed that decreased expression of NAc dopamine D2 receptors in human PCE offspring may contribute to such potentiated state (*71*). Future studies should examine whether this pharmacodynamic susceptibility, possibly related to impaired D2 autoreceptor function, can explain the specific increase in opioid reinforcing efficiency seen after maternal THC.

Dopamine release in the NAc gates striato-thalamic circuits, coupling internal states, exteroceptive stimuli, and motor invigoration mechanisms to ensure proper behavioral tuning to constantly evolving environments (*72*, *73*). Therefore, we asked whether the hyperdopaminergic and motivational disturbances associated with PCE were accompanied by changes in NAc neuronal patterns of encoding. We found that electrophysiological measurements matched dopamine findings, and a large bias toward outcome-predictive cues was determined. To explore the functional relationship of such neurobiological correlates with the behavioral phenotype observed, we opted for a machine learning–based approach. We decomposed the influence of PCE on NAc tensorial cell assemblies across declining rates of reinforcement (*27*), linking response vigor progression during PR with temporal encoding dynamics of outcome-predictive cues. Moreover, we observe that PCE progeny NAc neurons are more aligned with cue-triggered, effort-engendered patterns. Therefore, dopaminergic and striatal processing alterations identified in this study are notable contributors of the hypermotivational susceptibility conferred by in utero THC exposure. Notwithstanding, it must be noted that not all consequences of PCE on NAc dopamine release and neuronal encoding must derive from a unique, common alteration of VTA dopamine cell hyperexcitability (*12*). Unlike NAc encoding patterns, PCE effects on dopamine release were not modulated by decreasing rates of opportunity costs. This points to additional, overlapping ventral striatal modifications subsequent to PCE, which expand others described in the prefrontal cortex (*74*, *75*), and hippocampus (*76*).

Much like seen in human cohorts (*11*), the risk for augmented drug reinforcement was higher for males. Of note, sex-dependent behavioral effects of PCE paralleled its neurobiological signatures. That is, in those instances in which the behavioral consequences were sex specific (remifentanil PR rewards earned), dopamine release, and cue-evoked neuronal firing rates were also found exclusively up-regulated in males. This phenomenon is consistent with sex-specific shifts in excitatory-to-inhibitory synaptic balance observed in VTA dopamine cells (*14*), as well as NAc epigenetic alterations observed following PCE in males but not in females (*17*).

Although we present an interpretative framework for addiction-related vulnerabilities, the causative mechanisms linking THC-triggered molecular cascades in the developing brain to the enduring neurobiological signatures identified here remain largely unknown. The comprehensive study of Tortoriello *et al.* (*77*), however, suggests that fetal THC destabilizes microtubule organization in developing neurons and dampens CB1R influence in the assembly of fetal brain circuits (*78*). It remains to be seen how fetal sex could interact with the affected substrates to promote male-specific vulnerability observed across neurobehavioral domains and species. In addition, previous reports found that females were more vulnerable to the motivational consequences of perinatal THC exposure (*79*, *80*). These studies used higher-dose THC treatments (5 mg/kg), which continued up until pup weaning. This raises the possibility of different yet-to-be studied sex-modulated mechanisms that may give rise to vulnerability states either in males or females. Sex-dimorphic maturation time courses for synapse formation and pruning of dopaminergic neurons, as well as different CB1R receptor reserve levels between sexes on dopamine neuron axon cone growth may explain why developmental THC could affect males or females differently, depending on the dose and duration of exposure.

In conclusion, the persistent striatal processing and behavioral modifications identified here highlight a notable public health concern. The spurious neurodevelopmental effects of PCE accompany the propensity for late-onset motivational disturbances and potentially drug addiction issues, underscoring an urgent need for enhanced awareness and mitigation strategies regarding the use of cannabis during pregnancy.

## MATERIALS AND METHODS

All experimental procedures conformed to the National Institute of Health *Guide for the Care and Use of Laboratory Animals.* Ethical approval was granted by the Institutional Animal Use and Care Committee at the University of Maryland, Baltimore (IACUC protocol no. 00000054). The ARRIVE guidelines for reporting animal research have been followed.

### Animals

For all experiments, male and female Sprague-Dawley rats (Charles River) were used. Animals were housed in a temperature- and humidity-controlled room (24°C and 40 to 50% humidity, respectively) and maintained on a 12-hour light/dark cycle (07:00 to 19:00). All experiments were conducted in the light cycle. The number of rats for each experiment are indicated in the respective figure legends. Sex of the animals is reported throughout the figure legends. For all experimental measures, subjects from at least four different THC-exposed liters were included. All experimenters were blind to treatment assignments once liters were weaned at PND21.

### Prenatal THC exposure

Primiparous female Sprague-Dawley rats (Jackson Laboratories), mated with males, were used as maternal subjects, and housed individually during pregnancy. Each day, from GD5 to GD20, they were administered either THC (2 mg/kg and 2 ml/kg, s.c.) or vehicle. THC [dissolved in an ethanol solution (100 mg/ml)] was obtained from National Institute on Drug Abuse Drug Supply Program. Then, it was emulsified in 2% Tween 80, sonicated for 5 min and dissolved in sterile physiological saline. This specific dose was selected because it has been shown to not induce the typical behavioral responses observed in the cannabinoid tetrad assay, nor does it lead to cannabinoid tolerance (*81*). Previous studies by our group have demonstrated that this dose does not significantly affect maternal behavior, behavioral nonmaternal activity, or the body weight of the offspring (*13*, *14*). This dose is also comparable to the THC concentration found in mild cannabis joints (5%) and reflects moderate human cannabis consumption levels (*82*).

Offspring were weaned at ~PND21 and maintained without any further manipulation with access to food and water until experimental day (PND90). The study’s overall design was as follows: Two independent cohorts of control and PCE subjects were used, one subjected to food-seeking operant training and the other to remifentanil self-administration. In each cohort, a portion of the subjects underwent fiber photometry surgery, another portion were implanted with multiple-electrode arrays, and a third portion did not undergo any NAc implantation procedure. No differences based on NAc implantation strategy were observed in terms of operant food or remifentanil seeking. Figures 1 and 5 comprise behavioral measurements from all implanted and nonimplanted rats. Data from the exponential demand (Fig. 8) and go/no-go tasks (Fig. 9) were obtained from the nonimplanted set rats.

### Apparatus

Rats were tested in operant chambers (12.0″ L × 9.5″ W × 8.25″ H, Med Associates) inside sound-attenuating cabinets. Each chamber was equipped with two retractable levers (located 2 cm above the floor) as well as one light-emitting diode (LED) stimulus light and a 2.5-kHz tone-generating speaker located above each lever. An external food magazine delivered food pellets to a dispenser centrally located between the two levers. Alternatively, remifentanil infusions were delivered in a 20-ml injection over 2 s via a syringe mounted on a microinfusion pump (PHM-100A, Med-Associates) through a single-channel liquid swivel (375/25, Instech Laboratories) connected via tygon tubing to the chronic indwelling catheter. A house light and a white-noise speaker (80 dB, masking noise background) were located on the opposite wall. Throughout all operant paradigms, a multisensorial combination of conditioned stimuli (lever retraction, light stimuli, and auditory cues) was used to avoid potential sensorial confounds imposed by PCE.

### Food self-administration

After 2 to 4 weeks of recovery and viral expression, or ~PND120, rats were food restricted to 85 to 90% of their original body weight and trained to lever press for palatable food pellets (Bio-Serv no. F0299) under an FR1 schedule of reinforcement. After nine 45-min daily sessions, rats progressed to an FR5 schedule, which continued for 4 days. Following FR5 training, a PR schedule of appetitive reinforcement was used to estimate the effort rats were willing to expend for a food reward. On each successive trial, the response requirement (lever presses) needed to obtain reward scaled near-logarithmically. The response ratio of the first 16 trials was as follows: 1, 2, 4, 6, 9, 12, 15, 20, 25, 32, 40, 50, 62, 77, 95, and 118. The last response requirement attained, also known as breakpoint, was recorded and used to infer the inherent motivation for the reward. The PR session ended whenever no reward could be obtained within 10 min. During FR1, FR5, and PR training, animals had continued access to the active and inactive levers (except for the timeout period, 2 s) and each active lever press was rewarded with a single pellet. Responses on the inactive lever were recorded but had no programmed consequences.

### Exponential food demand task

Following PR testing, a subset of food-trained rats were tested on five 50-min demand task sessions. Each session was divided into five 10-min bins in which the lever pressing requirement for earning a food pellet followed a descending trend: 180, 90, 45, 15, and 5. We used a descending unit price to prevent excessive food intake and satiation during the beginning of the session. We recorded the number of pellets in each bin (averaged across sessions) as the primary dependent measure during this experiment. Inactive lever presses had no consequences. We fitted the data to the exponential model of demand equation (*45*)

$$\text{log}\;Q=\text{log}\;Q_{0}+k\left(e^{-\alpha \left(Q_{0}\cdot \text{price}\right)}-1\right)$$

Where *Q*_0_ is maximal demand at zero cost, *k* is a shared scaling constant that reflects the range of the data, α determines the rate of decline in relative consumption, and price is the independent variable of cost (responses required per pellet). We calculated essential value (EV) and *P*_max_ for each animal using the following formulas (*83*)

$$\text{EV}=1/\left(\alpha \cdot k^{1.5}\cdot 100\right)$$

$$P_{\text{max}}=1/\left(Q_{0}\cdot \alpha \cdot k^{1.5}\right)\cdot \left(0.083\cdot k+0.65\right)$$

### Go/no-go task

Following behavioral economics testing, rats started go/no-go pretraining. In this phase (three daily sessions), rats were only cued with go trials (cue light, 2.5-kHz tone), in which they were required to lever press within 5 s of its presentation to obtain a pellet reinforcer. If the 5 s elapsed with no response, the lever would retract, no reward would be presented, and a new trial would begin. Intertrial interval was on average 40 s, and each session consisted of 64 trials. Rats were then trained to respond to both go and no-go trials (white noise and house light). Thirty-two trials of each class were presented each session, and their order was pseudorandomized in blocks of 10 to 11 trials. Go and no-go trials were cued during 5 s. After 13 sessions, rats of both sexes progressed to phase 2 of go/no-go responding, in which all parameters were kept the same but no-go trials were extended to a 10-s duration.

### Intravenous remifentanil self-administration

After 2 to 4 weeks of recovery and viral expression, or ~PND115, rats were subjected to jugular vein catheterization surgery. Surgical implantation of the catheter was performed following anesthesia using isoflurane in O_2_ (4% induction and 2% maintenance). Indwelling intravenous silastic catheters (1.6 mm outer diameter) (Instech no. C30PU-RJV1611) were implanted 3.8 cm into the right jugular vein and anchored with suture. The remaining tubing ran subcutaneously to the 22-gauge vascular access button (Instech no. VABR1B/22), which exited at the midscapular region and remained protected with a magnetic aluminum cap (Instech no. VABRC). All incisions were sutured and coated with antibiotic ointment (Bactroban, GlaxoSmithKline). After surgery, animals were allowed to recover for 8 to 5 days before initiation of self-administration sessions. To maintain patency, catheters were flushed daily with heparinized saline (0.18 mg/ml). After surgery recovery, rats were trained (FR1) to lever press for remifentanil infusions (0.9 μg/kg in 20-μl injection over 2 s) through a single-channel liquid swivel (Instech Laboratories no. 375/22PS) connected via tygon tubing to the chronic indwelling catheter. After seven 2-hour-long sessions, response requirements increased (FR5) and testing continued for five (2-hour) sessions more. Each infusion was followed by a 10-s timeout period in which a nosepoke on the active hole had no consequences but was recorded. Then, rats were tested in a PR schedule for three daily sessions (as previously explained). After each experimental phase transition, rats showing marked signs of body weight loss or distress were removed from the study.

#### Extinction and cue-induced reinstatement

Following remifentanil intake phases, rats underwent operant extinction of drug-seeking behavior. Extinction sessions (2 hours long, once daily) were conducted for a minimum of 7 days and active lever presses produced neither drug delivery nor cue presentation. When animals showed less than 30 lever presses for two consecutive extinction sessions, they were reintroduced to the drug-associated compound cue. The cue-induced reinstatement sessions (FR1) lasted 2 hours and lever presses resulted in cue presentation and microinfusion pump activation but not remifentanil infusion.

### Exponential remifentanil demand task

Following PR responding, a subset of remifentanil-trained rats were tested on an exponential remifentanil demand task. All training and modeling parameters were the same as in the food demand task, with the exception of the pressing requirement progression, which was adjusted to: 6, 10, 16, 25, 40, 63, 100, and 158 (*84*) and resulted in 80-min sessions.

### Fiber photometry

Four weeks before their respective operant training procedures started, food- and remifentanil-seeking rats were subjected to chronic optical fiber implantation surgery. Rats were anaesthetized with isoflurane in O_2_ and then placed in the stereotaxic apparatus. The viral vector AAV9-hSyn-DA-sensor 4.4 (GrabDA_2m_) (1 μl, 10^13^ gc/ml) was unilaterally injected in the NAc using the following coordinates: anteroposterior, +1.3 mm; mediolateral, ±1.4 mm; and dorsoventral, −7.2 mm, relative to bregma (skull surface). Immediately following virus infusion, an optical fiber (diameter, 400 μm; numerical aperture, 0.5; Thorlabs) embedded within a ceramic ferrule was implanted with its tip targeting 0.1 mm above the abovementioned NAc DV coordinates. Fiber photometry of GrabDA_2m_ signals was conducted in all PR sessions. Two fiber-coupled LEDs producing 470- and 405-nm lasers [Lx465, Lx405; Tucker-Davis Technologies (TDT] were used as the excitation source. The LED beams were reflected and coupled to a fluorescence minicube (FMC4, Doric Lenses). A 2-m-long optical fiber (400 μm, Doric Lenses) was used to transmit light between the fluorescence minicube and the implanted fiber. A RZ10x real-time processor (TDT) was used to convert the current signal to voltage signal, which was processed through a low-pass filter (6 Hz, sixth-order Butterworth filter) built-in to the Synapse software (TDT). GrabDA_2m_ signals were processed using the MATLAB script developed by Barker *et al.* (*85*). Noise-related changes in fluorescence across the whole experimental session were removed by scaling the isosbestic control signal (405 nm) and regressing it onto the dopamine-sensitive signal (470 nm). *z*-scored PSTHs were obtained after each PR ratio completion (baseline −4 to −2 s, relative to cue onset). Alternatively, spontaneous GrabDA_2m_ transients before and after the remifentanil (10 μg/kg, i.v.) injection test were identified using the “findpeaks” (min peak prominence = 1) MATLAB function.

### In vivo electrophysiology

Two weeks before food or remifentanil operant training, rats underwent surgical implantation of an 8 or 16 microwire array. Electrodes were made of Teflon-insulated stainless steel (0.25-mm interelectrode space and 0.5-mm inter-row space; MicroProbe). Electrodes were fixed to the skull with acrylic cement and stainless steel bone screws. A stainless steel wire accompanying each array served as a ground electrode and was inserted into the midbrain/cerebellum. The Multi-channel Acquisition Processor (Plexon Inc.) was used for in vivo electrophysiology recordings. Headstages for either 8 or 16 electrodes with 1× gain and 36-gauge wire cables were used. A second level of amplification occurred at a 16-channel differential preamplifier with a fixed gain of 100×. Further amplification was performed through the online sorter software (gain, 1000 to 32000), filtering with a 500 Hz highpass cut-off and a bandpass filter between 0.7 and 300 Hz. Thresholds for each channel were chosen by the experimenter and typically one to five units were recorded per channel, excluding the reference channel. Voltage boxes were used to sort spikes within a channel. Activity was recorded for a 3-min baseline before the start of behavioral paradigms and terminated at the completion of the task. Offline spike sorting was performed in Offline Sorter (Plexon Inc) based on PCA and visual inspection of waveforms. After the spike-sorting procedure, the firing rate of each neuron was normalized to the average firing rate for that neuron during the entire session. Normalized firing rates were then standardized (*z*-scored) across units. After normalization, a Gaussian kernel sliding window of eight bins (σ = 3) was applied (“gaussfilter” MATLAB function). Distinct NAc patterns of encoding during PR were uncovered using supervised *k*-means clustering. The features extracted for clustering were as follows: (i) number of transients (peaks and troughs), (ii) time to first transient event relative to cue onset, (iii) mean activity during cue presentation, and (iv) mean activity after reward delivery. *k* values (number of clusters) were iteratively increased as long as new encoding patterns emerged. The final *k* value corresponded to the iteration before finding that the new patterns discovered resembled those already existing (*k* = 4 for the food PR dataset and *k* = 3 for the remifentanil PR dataset). Neurons that did not cluster into any pattern were considered as “nonencoding.” Mean-normalized firing rates (AUCz) were obtained using the AUC (“trapz” MATLAB function) during the corresponding analysis window (cue, 0 to 2 s; reward, 2 to 7 s; relative to cue onset, except otherwise stated).

### Tensor component analysis

To find a compact and interpretable description of the multitrial neural activity obtained from the PR task, we perform a dimensionality reduction technique called canonical polyadic (CP) tensor decomposition (*86*). CP is a generalization of PCA to higher-order data arrays (tensors). In this case, we fitted the dimensions *U × T × P*, where *U* are the individual recorded units, *T* are PR completed trials, and *P* is the firing rate PSTH around each cue presentation. On the basis of Williams *et al.* (*27*) study and MATLAB package (https://github.com/ahwillia/tensortools) we used the alternating least-squares (ALS) algorithm to iteratively optimize dimensionality reduction solutions until converging to minimal reconstruction errors. ALS optimizes one of the factors (e.g., the unit factor *W_u_*) while fixing the other two (the PSTH and trial factors *A_p_* and *B_t_*, respectively), yielding the following update rule

W←argminW~∑utp(Xutp−∑rw~urbtrapr)2

This procedure is then cyclically applied by optimizing one of the other factors and fixing the remaining two. Fitting TCA to data is a nonconvex problem (*27*). Thus, the model was fitted multiple times, each time starting from a different random initial parameter set, and the number of components, here referred to as “cell assemblies,” was gradually adjusted until the smallest number of recovered components (*r* = 4) that converged across multiple runs was found. These low-dimensional four cell assemblies largely resembled those recovered by traditional *k*-means clustering. To ensure that all arrays (dimensions) were of the same length, only data from the first eight PR trials (lowest reward earned on that session) was included. Functional relationships between uncovered cell assemblies with response vigor during PR was examined using a machine learning prediction algorithm. A gradient boosted trees model was trained on each neuron unit factors *W_u_* (80% data hold for the training set) to predict latency to eighth PR reward (TensorFlow, Google). The validity of this prediction was determined by correlating ground truth values with predicted latencies (Pearson’s *r*).

### Histology

Rats underwent isoflurane anesthesia (5%) and transcardial perfusion with a 4% paraformaldehyde (PFA) solution in a 0.1 M sodium phosphate buffer (PB) at pH 7.4. Following perfusion, brains were postfixed at 4°C in PFA overnight. Brain sections (40 μm thick) were obtained using a vibratome (Leica). Coronal sections were immersed in a PB solution containing 3% normal donkey serum (Jackson 017-000-121) for a duration of 30 min. Immunodetection of GrabDA_2m_ was achieved following 2-hour incubation with an anti–green fluorescent protein antibody (Thermo Fisher Scientific no. A-11122, RRID AB_221569) (1:200) and labeled with goat anti-rabbit secondary antibody Alexa Fluor Plus 555 (Thermo Fisher Scientific no. A32732, AB_2633281) (1:2000, 45 min).

### Statistical analyses

Parametric behavioral, dopaminergic, and firing rate (AUCz) measures were analyzed using a one- or two-way repeated-measures (RM) analysis of variance (ANOVA), or unpaired two-tailed Student’s *t* tests and Bonferroni post hoc tests were used to correct for multiple comparisons when appropriate. Every time samples were split into different groups, such as sexes, following Student’s *t* tests were corrected for multiple comparisons using the Bonferroni-Dunn method. Whenever violations of homoscedasticity were detected, a Welch correction was applied (Welch’s *t* test). When analyzing nonparametric variables (proportion of subjects), the χ^2^ tests were used. Significance was set at *P* < 0.05 and all tests were two tailed. To determine biases in the assignment of neurons corresponding to the identified clusters (Figs. 3 and 7), a Bayesian logistic regression model using a compound confluent hypergeometric prior distribution (α = 0.5, β = 2, and *s* = 0) was constructed. Statistical analyses were performed in MATLAB 2023b, GraphPad Prism 10.1, and JASP 0.18. Data describe biological replicates. Each behavioral finding was replicated in at least three different cohorts. No statistical methods were used to predetermine sample sizes. Dam treatment assignment was random and experiments were blind to the subjects’ history of prenatal exposure.
