# Supplementary material for: Dynamic overrepresentation of accumbal cues in food- and opioid-seeking rats after prenatal THC exposure
Source: Sci Adv. 2024 Nov 8;10(45):eadq5652. doi: 10.1126/sciadv.adq5652 (PMC11546747; doi:10.1126/sciadv.adq5652)
Supplement: Supplementary file 1 — Figs. S1 to S5 [file sciadv.adq5652_sm.pdf]

Science Advances
AAAS

Supplementary Materials for

Dynamic overrepresentation of accumbal cues in food- and opioid-seeking rats after prenatal THC exposure

Miguel Á. Luján *et al.*

Corresponding author: Miguel Á. Luján, mlujan@som.umaryland.edu

Sci. Adv. **10**, eadq5652 (2024)
DOI: 10.1126/sciadv.adq5652

This PDF file includes:

Figs. S1 to S5

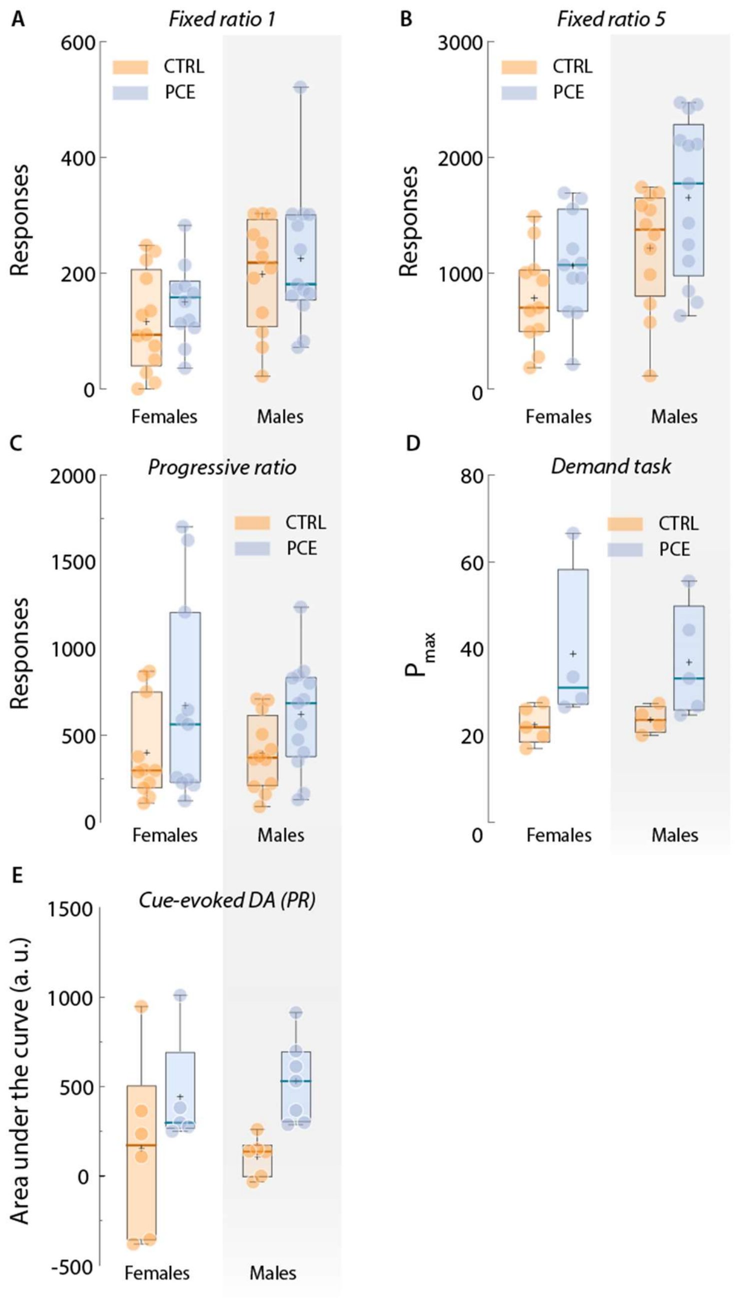

Fig. S1. Lack of sex-dependent effects of PCE on motivation for food rewards. A) Total lever presses under a FR1 schedule of reinforcement (females; $t_{22} = 0.99$, Bonferroni-Dunn-corrected $p = 0.66$) (males; $t_{23} = 0.54$, Bonferroni-Dunn-corrected $p > 0.99$). nCTRL = 25 (13F, 12M); nPCE = 24 (11F, 13M). **B)** Total lever presses on FR5 (females; $t_{20} = 1.49$, Bonferroni-Dunn-corrected $p = 0.30$) (males; $t_{23} = 1.78$, Bonferroni-Dunn-corrected $p = 0.17$). nCTRL = 23 (11F, 12M); nPCE = 24 (11F, 13M). **C)** Total responses on PR testing (females; $t_{20} = 1.40$, Bonferroni-Dunn-corrected $p = 0.17$) (males; $t_{22} = 2.07$, Bonferroni-Dunn-corrected $p = 0.050$). nCTRL = 23 (11F, 12M); nPCE = 24 (11F, 13M). **D)** P_{max} values derived from the food demand task (females; $t_7 = 1.91$, Bonferroni-Dunn-corrected $p = 0.19$) (males; $t_7 = 1.98$, Bonferroni-Dunn-corrected $p = 0.17$). nCTRL = 9 (5F, 4M); nPCE = 9 (4F, 5M). **E)** PCE increases cue-evoked NAc dopamine release in a sex-independent manner (2-way ANOVA; $^{sex*treatment}F_{1,20} = 0.24$, $p = 0.62$; $^{treatment}F_{1,20} = 7.41$, $*p = 0.013$). Center line represents the median; the cross illustrates the average; the bounds of the box depict the 25th to 75th percentile interval; and the whiskers represent minima and maxima.

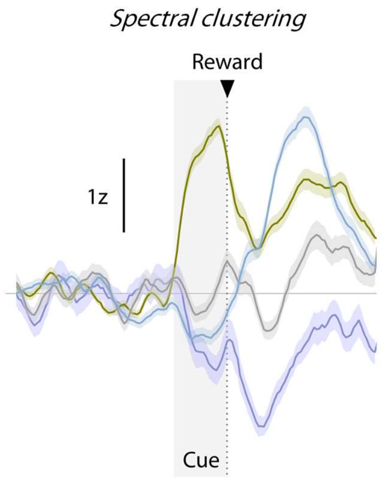

Fig. S2. NAc patterns of encoding identified by spectral clustering. Trial-averaged, cue-aligned PSTH of the four neuronal clusters uncovered by spectral decomposition. Lines represent average values and shaded areas ± SEM values.

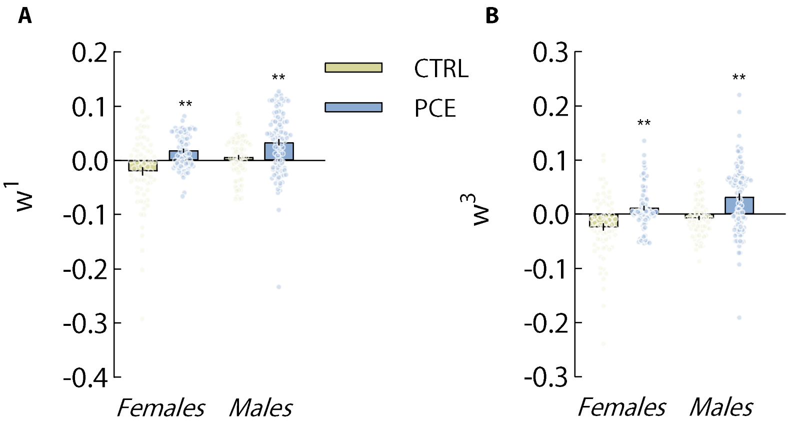

Fig. S3. PCE effects on cue-encoding tensorial assemblies are not sex-dependent. A) Individual unit factor loadings (w^1) of the cue-activated tensorial cell assembly ($a^1{}_p b^1{}_t w^1{}_b$) identified by TCA (females; $t_{143} = 4.43$, Bonferroni-Dunn-corrected $^{**}p < 0.001$) (males; $t_{172} = 3.50$, Bonferroni-Dunn-corrected $^{**}p = 0.001$). **B)** Individual unit factor loadings (w^3) of the cue-inhibited tensorial cell assembly ($a^3{}_p b^3{}_t w^3{}_b$) (females; $t_{143} = 4.21$, Bonferroni-Dunn-corrected $^{**}p < 0.001$) (males; $t_{172} = 4.78$, Bonferroni-Dunn-corrected $^{**}p < 0.001$). $n_{\mathrm{CTRL}} = 143$ (68F, 75M); $n_{\mathrm{PCE}} = 176$ (77F, 99M). For all bar and point plots, bars denote mean ± SEM.

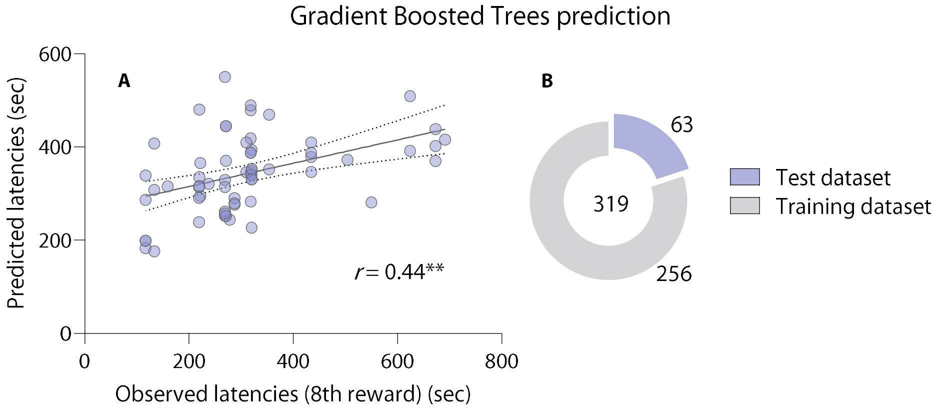

Fig. S4. Tensorial unit loadings predict latency to reward during PR responding. A) Correlation between observed and predicted latency to complete 8 trials during PR (*ground truth* vs. *predicted value*; Pearson's **$r = 0.44$, $p = 0.003$). **B**) A gradient boosted decision tree machine learning algorithm was trained on 80 % of the unit loadings and tested on the remaining 20 % cases.

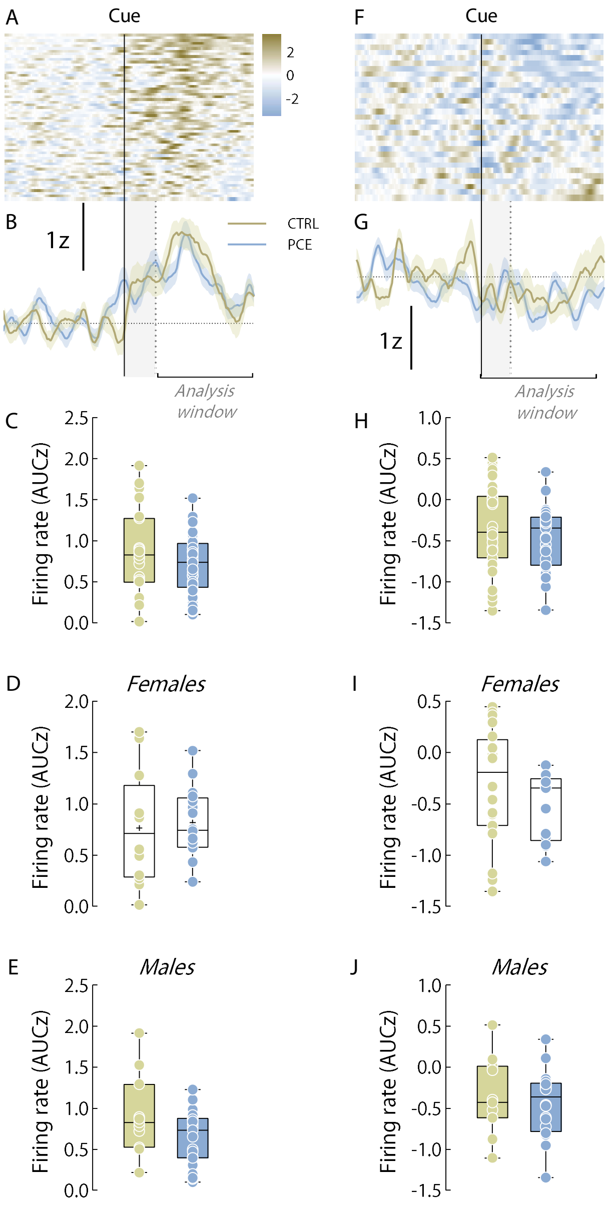

Fig. S5. Reward-responding and lever press-inhibited NAc neuronal clusters identified during remifentanil operant seeking. A) Trial-averaged heatmap depicting activity from all reward-responding units, centered around cue onset (vertical black line). **B)** Reward-responding neurons firing rate (Z-score). Shaded grey areas represent cue presentation and horizontal brackets indicate the time window used for parametric analyses. Lines represent average firing rates and colored shaded areas depict ± SEM. **C-E)** Trial-averaged firing rates (AUC) from the reward-activated cluster following remifentanil infusion (Welch's $t_{33.4}$= 1.03, p = 0.31) (females; t_{26} = 0.31, Bonferroni-Dunn-corrected p > 0.99) (males; t_{32} = 1.94, Bonferroni-Dunn-corrected p = 0.12). nCTRL = 23 (12F, 11M); nPCE = 34 (11F, 23M). **F-G)** Heatmap and line plots depicting averaged encoding pattern of the lever press-inhibited cluster. **H-J)** Trial-averaged firing rates (AUC) from the lever press-inhibition cluster preceding cue presentation (t_{62}= 1.27, p = 0.21) (females; t_{62} = 1.26, Bonferroni-Dunn-corrected p = 0.41) (males; t_{29} = 0.51, Bonferroni-Dunn-corrected p > 0.99). nCTRL = 42 (31F, 11M); nPCE = 53 (33F, 20M).
